# Scanning the Landscape of Genome Architecture of Non-O1 and Non-O139 *Vibrio cholerae* by Whole Genome Mapping Reveals Extensive Population Genetic Diversity

**DOI:** 10.1371/journal.pone.0120311

**Published:** 2015-03-20

**Authors:** Carol Chapman, Matthew Henry, Kimberly A. Bishop-Lilly, Joy Awosika, Adam Briska, Ryan N. Ptashkin, Trevor Wagner, Chythanya Rajanna, Hsinyi Tsang, Shannon L. Johnson, Vishwesh P. Mokashi, Patrick S. G. Chain, Shanmuga Sozhamannan

**Affiliations:** 1 Henry M. Jackson Foundation, Bethesda, Maryland, United States of America; 2 Naval Medical Research Center—Frederick, Fort Detrick, Maryland, United States of America; 3 OpGen, Inc., Gaithersburg, Maryland, United States of America; 4 University of Florida, Gainesville, Florida, United States of America; 5 Genome Science, Biosciences Division, Los Alamos National Laboratory, Los Alamos, New Mexico, United States of America; United States Army Medical Research Institute for Infectious Disease, UNITED STATES

## Abstract

Historically, cholera outbreaks have been linked to *V*. *cholerae* O1 serogroup strains or its derivatives of the O37 and O139 serogroups. A genomic study on the 2010 Haiti cholera outbreak strains highlighted the putative role of non O1/non-O139 *V*. *cholerae* in causing cholera and the lack of genomic sequences of such strains from around the world. Here we address these gaps by scanning a global collection of *V*. *cholerae* strains as a first step towards understanding the population genetic diversity and epidemic potential of non O1/non-O139 strains. Whole Genome Mapping (Optical Mapping) based bar coding produces a high resolution, ordered restriction map, depicting a complete view of the unique chromosomal architecture of an organism. To assess the genomic diversity of non-O1/non-O139 *V*. *cholerae*, we applied a Whole Genome Mapping strategy on a well-defined and geographically and temporally diverse strain collection, the Sakazaki serogroup type strains. Whole Genome Map data on 91 of the 206 serogroup type strains support the hypothesis that *V*. *cholerae* has an unprecedented genetic and genomic structural diversity. Interestingly, we discovered chromosomal fusions in two unusual strains that possess a single chromosome instead of the two chromosomes usually found in *V*. *cholerae*. We also found pervasive chromosomal rearrangements such as duplications and indels in many strains. The majority of *Vibrio* genome sequences currently in public databases are unfinished draft sequences. The Whole Genome Mapping approach presented here enables rapid screening of large strain collections to capture genomic complexities that would not have been otherwise revealed by unfinished draft genome sequencing and thus aids in assembling and finishing draft sequences of complex genomes. Furthermore, Whole Genome Mapping allows for prediction of novel *V*. *cholerae* non-O1/non-O139 strains that may have the potential to cause future cholera outbreaks.

## Introduction

Assessing the genetic diversity of a bacterial population aids in understanding the evolution of pathogenesis and the spread of virulence factors by horizontal gene transfer within that population. It also allows us to predict the potential of a given pathogenic clone to cause disease outbreaks. *Vibrio cholerae*, a Gram-negative bacterium and the causal organism of cholera, lends itself to such an inquiry since its virulence mechanisms are relatively well understood and there is a vast diversity of these organisms in the environment.

Cholera, a centuries-old disease, is responsible for an estimated 3 to 5 million acute diarrheal cases and 100 to 120 thousand deaths annually, even in modern times [1]. Cholera is endemic in parts of the world lacking adequate sanitary infrastructure and is a major concern during mass migration of populations due to natural or man-made disasters, such as the 2010 earthquake in Haiti [2]. In Haiti, since the beginning of the epidemic (October 2010) and until epidemiological week (EW) 23 of 2014, there have been 703,510 cholera cases, of which 393,912 were hospitalized (56%) and 8,562 died. The cumulative case-fatality rate remains 1.2%, with variations ranging from 4.4% in the Department of Sud Est to 0.6% in Port-au-Prince [3].

Immunodiagnosis of cholera is primarily based on the somatic O-antigen which is present on the bacterial surface and is the major protective antigen against *V*. *cholerae* infection [4]. There have been seven recorded cholera pandemics, all of which are associated with genetic variants of O1 serogroup, a group known as cholera *Vibrios*. Although the major virulence factors responsible for cholera such as cholera toxin, toxin coregulated pilus and ToxR present in O1 strains have also been shown to be present in many of the 205 other non-O1 serogroup strains [5], only two of the non-O1serogroups (O37 and O139) have been linked with cholera outbreaks in the past [6–7]. Thus, other factors present in *V*. *cholerae* O1 may play a role in its epidemicity.

Recent studies on Haiti cholera outbreak strains underscored the gap in recognizing the role of non-O1/non-O139 strains in cholera. Bacteriological analysis identified *V*. *cholerae* O1 and *V*. *cholerae* non-O1/non-O139 as sole pathogen in 48% and 21% of the samples, respectively. From the remaining 31% of the clinical samples, other enteric pathogens were cultured [8]. *V*. *cholerae* O1 and non-O1/non-O139 were co-cultured from 7% of the O1 positive samples. Combined with whole genome sequence data, the results suggested that two distinct *Vibrio* populations, *V*. *cholerae* O1 and *V*. *cholerae* non-O1/non-O139 may have contributed to the cholera epidemic in Haiti [8].

The Haiti study also brought the attention of the cholera research community to the critical need for an up-to-date, well curated and publicly available *Vibrio* reference genomic database that reflects global genetic diversity (strains from endemic and non-endemic regions) as well as phylogenetic diversity within cholera (O1/O139) and non-cholera (non-O1/non-O139) *Vibrios* [8]. The authors pointed out that such a qualified database is critically important if investigations of future *V*. *cholerae* epidemics are to be effective for attribution of source of the pathogen and timely for public health interventions [8]. The prevalence and contribution of non-O1/non-O139 strains to cholera in this study have been disputed; nonetheless, the presence of non-O1/non-O139 strains in Haiti cholera samples is duly acknowledged by various groups [8–10].

Whole genome sequencing efforts prior to the Haiti cholera study were focused primarily on epidemic strains: a survey of *V*. *cholerae* whole genome sequences present in public databases shows that more than 50% of sequences are that of O1 strains and about 20% are of non-O1/non-O139 strains while the rest are of unknown serogroups. Almost all the sequences are draft sequences and the nine complete or gapless sequences (as of Dec 2013) belong to O1 serogroup. Most of the sequenced non-O1/non-O139 strains are from the Haiti region thereby lacking geographic and temporal diversity [8].

In the present study two of the above described issues are addressed: firstly, assessing the global genetic diversity of *V*. *cholerae* using a genome scale approach and secondly, assessing the structural complexities in genomes and screening appropriate strains for complete or gap-less whole genome sequencing. Data presented here support the idea that high resolution whole genome restriction site based bar coding (Whole Genome Mapping) can be used successfully to assess the genetic diversity and structural complexity of a strain collection such as the non-O1/ non-O139 *V*. *cholerae*. Furthermore, data presented here also show extensive genomic rearrangements, such as indels and duplications, accounting for some of the large variations in genome sizes among *V*. *cholerae* strains. Interestingly, evidence is also presented for naturally occurring *V*. *cholerae* that possess single chromosomes as opposed to the traditional paradigm of dual chromosomes found in natural isolates of *V*. *cholerae* [11] or genetically engineered, laboratory-generated single chromosome containing *V*. *cholerae* strains reported in the past [12].

## Materials and Methods

### Bacterial Strains

**Fig 1:**
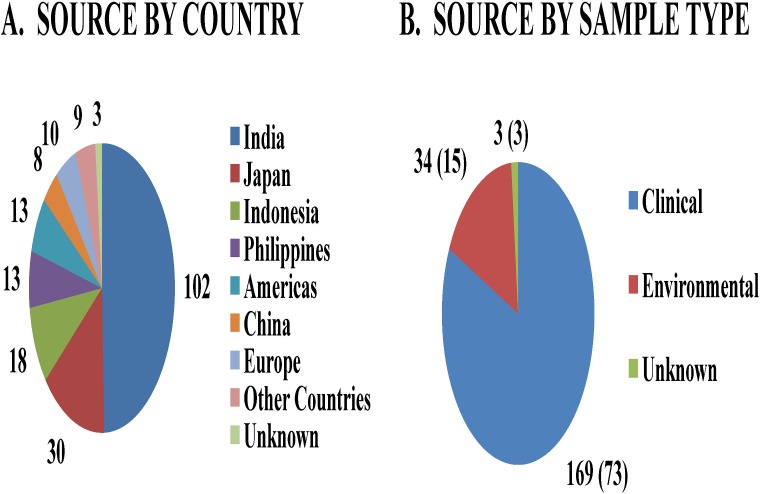
Strain collection features of the Sakazaki serogroup set. A) Breakdown of strains based on geographical location (country where the strains were isolated). B) Breakdown of strains based on isolation source. The number of strains for which Whole Genome Maps were generated in this study is indicated in parentheses.

The genetic diversity of *V*. *cholerae* as a species is not fully understood. Most genetic diversity studies focus on cholera *Vibrios*, while ignoring the pathogenic potential of non-cholera *Vibrios*; one notable exception is a recent study on Haiti cholera outbreaks [8]. A strain collection that captures the genetic diversity of *V*. *cholerae* is the Sakazaki serogroup cultures [13]. This collection is based on a bacterial classification scheme of the somatic O antigen and has 206 serogroups to date (S1 Table). These strains were collected from diverse sources from all over the world; a breakdown of the strain sources is shown in Fig. 1. Majority of the strains (167) were isolated from patients presenting non-cholera like diarrheal symptoms, two strains were from cholera cases, and 34 strains were isolated from environmental sources. Most of the strains were classified as *V*. *cholerae* while 9 *V*. *mimicus* strains were also placed in this collection. *V*. *cholerae* and *V*. *mimicus* appear indistinguishable by conventional serological methods. The H-antigen of *V*. *mimicus* is identical to that of *V*. *cholerae*, and the O-antigen groups of *V*. *mimicus* cross-react with a wide range of O-antigen groups of *V*. *cholerae*. Therefore, a single serotyping system has been in use for both species and as a result the *V*. *cholerae* serogrouping system contains *V*. *mimicus* as reference strains [13].

Although *V*. *mimicus* was previously recognized as a biotype of *V*. *cholerae*, it has now been reclassified as an independent species because of differences in a number of biochemical characteristics; e.g., *V*. *mimicus* is negative for sucrose fermentation, Voges-Proskauer test, lipase (corn oil) activity, and Jordan’s tartrate reaction [14,15]. The designations of *V*. *cholerae* strains in the Sakazaki serogroup set, the metadata pertaining to their collection, and those used for Whole Genome Mapping are presented in S1 Table.

### Whole Genome Mapping

The ARGUS system (Opgen, Inc, Gaithersburg, MD) was used for Whole Genome Mapping (Optical Mapping). Overnight bacterial cultures were grown from single colonies and genomic DNA was extracted according to a customized protocol provided by Opgen, Inc. Whole Genome Mapping was performed according to manufacturer’s instructions and the MapSolver software v3.2.2 (OpGen, Inc.) was utilized for subsequent analysis of map data. Whole Genome Map (WGM) was produced for each strain using *Nhe*I and with additional enzymes for some strains. The collected restriction map data were used to generate the final map assembly, estimate genome sizes and perform map comparisons. The whole genome map (WGM) data are available for download as cluster.xml files (S1 Dataset and S2 Dataset for Chr I and Chr II respectively). The estimated sizes of Chr I and Chr II are also presented in S1 Table. Methods for construction of similarity cluster, calculation of distance matrix and creation of dendrogram based on distance matrix by ARGUS Map Solver software have been published elsewhere [16]. Briefly, to construct the similarity cluster, each pair of maps is aligned using a dynamic programming algorithm based upon published methods [17–19]. This method finds the optimal alignment of two restriction maps according to a scoring model that incorporates fragment sizing errors, false and missing cuts, and missing small fragments. For a given alignment, the score is proportional to the log of the length of the alignment, penalized by the differences between the two maps, such that longer, better-matching alignments will have higher scores. This method has been used before to compare *E*. *coli* genomes [20]. From these alignments, adding up the lengths of the unmatched regions from both maps and dividing this by the sum of the lengths of both maps in the pair produces a dissimilarity score for a pair of maps. A matrix of these pairwise scores is used as input to Agnes, an agglomerative clustering method implemented in the R statistical package, which creates dendrograms using the unweighted pair group method with arithmetic mean (UPGMA). An analogous clustering method using sequence information has been shown to produce trees that match existing phylogeny data [21]; however, no specific evolutionary claims based upon the trees are made in this study.

### Pulse Field Gel Electrophoresis (PFGE)

Chromosomal DNA extraction and pulse field gel electrophoresis (PFGE) of *V*. *cholerae* N16961 (O1), 1154–74 (O49) and 10432–62 (O27) strains to estimate genome sizes were carried out according to published protocols with minor modifications [11,22]. Briefly, bacteria were grown in Luria-Bertani (LB) broth at 37°C with shaking at 200 rpm to a cell density of 0.8–1.0 at OD_600_. Chloramphenicol (180 μg /ml) was added and the culture was incubated for 1hour to arrest DNA replication and synchronize the cells. Ten mls of bacterial cells were pelleted and then re-suspended in the same volume of cell suspension buffer (CSB; 100 mM Tris: 100 mM EDTA [pH 8.0]) to retain the cell density at 0.8–1.0 of OD_600_. An aliquot (400 μls) of the cell suspension was transferred to an 1.5 ml centrifuge tube and mixed with an equal volume of molten 1.0% SeaKem Gold (SKG, Cambrex, Rockland, ME) agarose by gently pipetting up and down several times and the mixtures were immediately dispensed into the wells of a disposable plug mold (Bio-Rad Laboratories, Hercules, CA). The agarose plugs were allowed to solidify for 10–15 min at room temperature and then treated with lysozyme (5 mg/ml) and RNase (5 μg/ml) and processed as described [22]. After lysozyme treatment, the agarose plugs were either treated with restriction enzyme I-*Ceu*I or directly electrophoresed without restriction digestion. Electrophoresis was carried out in a CHEF-DR II gel apparatus in 0.5x TBE buffer at 4°C. The following pulse ramps were used: 60 to 90 sec for 60 hr at 200 V in 1.3% agarose. Agarose plugs containing seven *Hansenula wingei* chromosomes ranging in size from 1–3.1 Mb (Bio-Rad Laboratories, Hercules, CA) were used as molecular weight markers.

## Results and Discussion

### Whole Genome Mapping of non-O1/non-O139 *V*. *cholerae* strains reveals extensive genetic diversity

A Whole Genome Mapping approach was used to assess the genetic diversity of a fraction of the Sakazaki type culture collection (87 *V*. *cholerae* and 4 *V*. *mimicus* strains reported in this study). The WGM data are provided here as cluster.xml files for use in other studies if desired (S1 Dataset and S2 Dataset). The average estimated genome sizes based on WGM data for Chr I and Chr II were 2,981,226 bps and 1,095,858 bps with a maximum variability of 20% and 37% respectively. Thus, the variation in size of Chr II is much more pronounced than for Chr I (Table 1). By comparison, the published complete sequences of *V*. *cholerae* strains of the O1 serogroup are less variable in size. Among the nine finished genome sequences in the GenBank microbial genome database (as of Dec 2013), the calculated average sizes of Chr I and Chr II are 2,991,480 bps and 1,059,789 bps with a maximum variability of 12% and 16% respectively (Table 1). The limited variability in O1genome sizes may reflect the clonal nature of the O1strains as opposed to the non-O1/non-O139 strains.

**Table 1:** Genome size estimates based on whole genome mapping compared to whole genome sequencing.

Category	Total	Chr	Average (bps)	SD (bps)	Range (bps)	Maximum Variability (%)
WGM*	89	I	2,981,226	103,782	2,659,377–3,268,280	20.4
Increase#	75	I	_	_	1,643–375,757	12.6
Decrease#	14	I	_	_	4,839–233,146	7.8
WGM*	89	II	1,095,858	85,035	958,630–1,358,831	36.5
Increase #	61	II	_	_	4,634–312,449	28.5
Decrease #	28	II	_	_	255–87,752	8
	O27	I/II	3,801,481	_	_	_
	O49	I/II	3,889,393	_	_	_
WGS**	9	I	2,991,480	101,285	2,791,729–3,149,584	11.9
		II	1,059,789	49,071	946,986–1,111,222	15.5

SD-Standard Deviation;

*WGM-Whole Genome Map (Optical maps);

**WGS-Whole Genome Sequences in NCBI database(finished genomes and all O1 strains);

# size compared to M66–2 whole genome sequence

**Fig 2:**
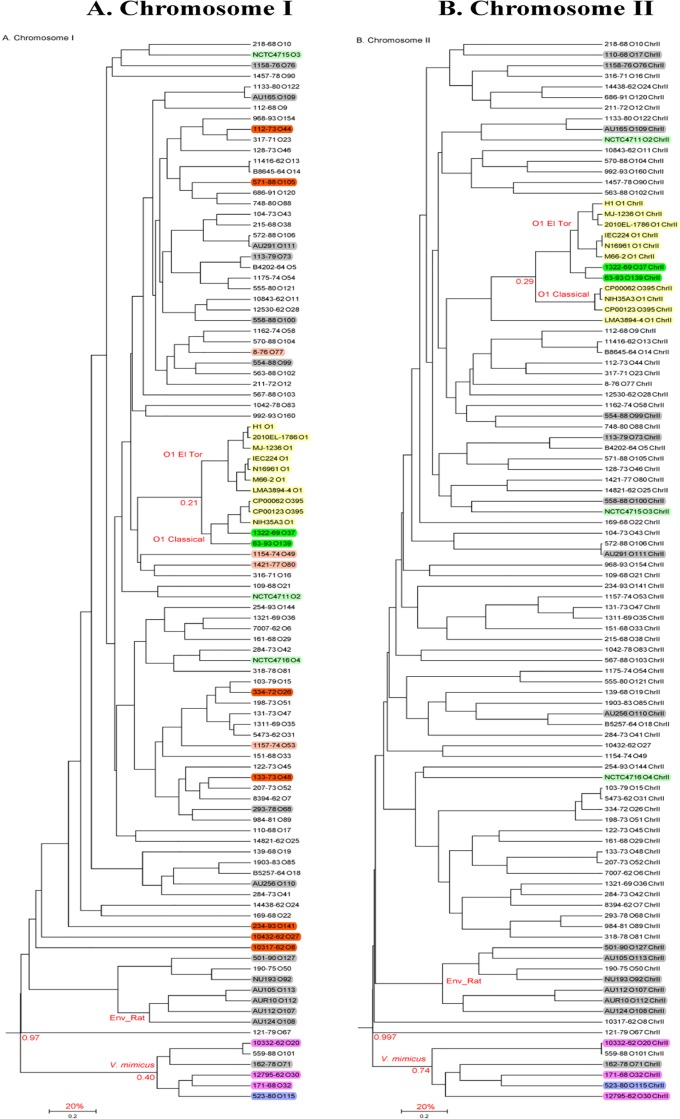
UPGMA method based dendrograms of Sakazaki serogroup strains. Whole Genome Mapping data based distance matrix with default parameters was used to generate the dendrograms. The three clusters (epidemic *V*. *cholerae* Classical and El Tor, one group of environmental isolates from rat, and *V*. *mimicus*) are indicated on the branches. The distribution of VPI and CTX on chromosome I in non-O1/non-O139 strains are indicated as well. The different colors of the highlighted strains indicate the following characteristics: No highlight- Clinical; Gray-environmental; Light green (serogroups O2-O4) source unknown; Yellow: epidemic O1 Classical and El Tor; Green: non-O1 epidemic strains; Pink: *V*. *mimicus*; Blue: One *V*. *mimicus* isolate (serogroup O115) that has the VPI cluster; Orange: Clinical and carry both VPI and CTX clusters; Light red (O77, O49, O80, O53): Clinical and carry VPI only. Scale: 0.2 = 20% dissimilarity.

OpGen MapSolver v3.2.2 and UPGMA method (default parameters) were used to independently cluster strains based on restriction maps of Chr I and Chr II. The Whole Genome Mapping based dendrograms (Fig. 2) revealed extensive diversity among strains in this collection, as evidenced by the large number of clades and very few major clusters with the exception of three minor clusters: (a) epidemic cluster that comprised of the O1 Classical and El Tor and (b) an environmental cluster comprised of strains from the same environmental source (rat) and (c) *V*. *mimicus*. As expected, the *V*. *mimicus* cluster is an outlier as it is quite removed from the rest of the *V*. *cholerae* strains (dissimilarity index of approximately 97%). Whole Genome Maps of all the 91 strains revealed dissimilarity index ranging from 20%-97% based on restriction site distance matrix. The percentage dissimilarities (denoted as a fraction of 1) are indicated at some interesting cluster nodes of the dendrograms (Fig. 2). Similar to these results, in an MLST study, 66 sequence types among 77 strains with only 3 clonal complexes were found (CC1–7 sequence types (STs); CC2 and CC3 represented by 2 STs each) [23]. Bayesian algorithm based STRUCTURE [24] analysis of the MLST data set of 77 strains identified four subpopulations; clinical strains were found predominantly in subpopulations I and III [23].

The clonal nature of the epidemic strains is clear; as shown in previous studies, strains that cause cholera epidemics, or have epidemic potential, cluster together. The epidemic cluster not only contains the O1 type strains but also the O139 and O37 strains known to have caused cholera outbreaks in the past, supporting earlier conclusions [5,6,25]. The O139 and O37 strains possess the genetic backbone of the O1 El Tor and O1 Classical strains, respectively, with the O139 and O37 O-antigen gene clusters acquired via horizontal gene transfer [6,25]. Interestingly, unlike an earlier study [25] which placed O37 closer to the classical O1 and O139 closer to the O1 El Tor strains based on MLST and RFLP of virulence regions, Whole Genome Mapping based clustering of Chr I placed both O139 and O37 with Classical strains and that of Chr II clustered with El Tor strains. In addition, many strains known to possess some of the major virulence factors are scattered throughout the tree, supporting the role of horizontal gene transfer in dissemination of these genes (Fig. 2). In addition, no additional serogroup strains were found to cluster in this branch ruling out the possibility of the existence of non-O1/non-O139 strains with epidemic potential in the strain collection examined here.

### Comparative Whole Genome Map analysis and identification of genomic variations: identification of strains with chromosomal fusions

**Fig 3:**
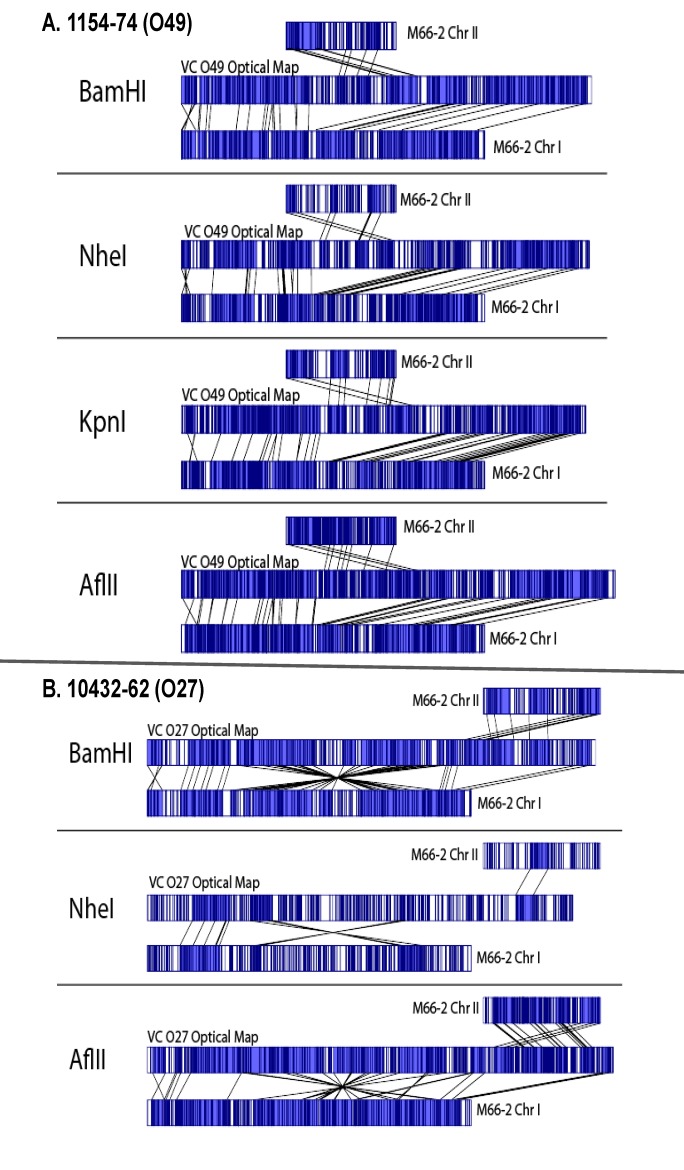
Whole Genome Mapping using different restriction enzymes supports the Chr I and Chr II fusions in *V*. *cholerae* 1154–74 (O49) and 10432–62 (O27) strains. A) The top four panels of maps are derived from strain 1154–74 (O49) and B) the bottom 3 panels are from strain 10432–61 (O27). In each panel, the top and bottom maps indicate *in silico* generated Chr II and Chr I restriction maps of M66–2 sequences respectively, compared to the experimentally generated maps (middle) of 1154–74 or 10432–62 using indicated restriction enzymes. The fusion in 1154–74 (O49) is around 1.29 Mb of Chr I (size 2.89 Mb) to 0.32 Mb of Chr II (size 1.05) of M66–2. The fusion in 10432–62 (O27) has occurred around 2.80 Mb of Chr I (2.89 Mb) to around 0.83 Mb of Chr II (1.05 Mb) in M66–2. The blue region indicates a single copy match between the chromosomes compared.

Whole Genome Map comparisons were performed to identify genome wide DNA rearrangements. Interestingly, among the 91 strains analyzed by Whole Genome Mapping, two strains, 1154–74 (O49) and 10432–62 (O27) were found to possess a single chromosome instead of the two chromosomes found in the rest of the panel. Examination of individual DNA molecules spanning the fusion junctions (data not shown) strongly suggested that this fusion is real and not an artifact of spurious chimeras of uncut restriction fragments. Initially, Whole Genome Maps were generated using *Bam*HI enzyme. Comparative genome analysis of the *Bam*HI Whole Genome Map of *V*. *cholerae* 1154–74 (O49) to *in silico* generated reference maps of strain M66–2 (GenBank accession numbers for Chr I and Chr II: CP001233.1 and CP001234.1 respectively), indicated that the two chromosomes have been fused in this strain (Fig. 3A). In order to further validate the single chromosome finding, additional enzymes (*Nhe*I, *Kpn*I and *Afl*II) were used to generate Whole Genome Maps of the *V*. *cholerae* strain 1154–74 (O49) and these maps were compared to that of M66–2. In all these cases, fusion of Chr I and Chr II in *V*. *cholerae* strain 1154–74 (O49) was evident. Based on Whole Genome Map data, the estimated locations of the fusion on Chr I start at 1,269,909 bps and end at 1,281,727 bps (11,818 bps of apparent net deletion) and on Chr II start at 323,846 bps and end at 418,779 bps (94,933 bps of apparent net deletion) of the reference genome coordinates (Fig. 3A).

Similarly, strain 10432–62 (O27), also displayed a fusion between Chr I and Chr II. This fusion was confirmed by Whole Genome Maps generated using three different enzymes: *Bam*HI, *Nhe*I, and *Afl*II (Fig. 3B). However, in this case, the fusion junctions were different from that of *V*. *cholerae* 1154–74 (O49). The estimated locations of the merge on Chr I start at 2,796,629 bps and end at 2,725,724 (70,905 bp of apparent net deletion) and on Chr II start at 822,160 and end at 832,832 bps (10,672 bp of apparent net deletion) of the reference genome coordinates (Fig. 3B).

The fusion junctions in both strains have large repeats containing IS elements and/or prophages indicating potential recombination cross-over regions between Chr I and Chr II (unpublished whole genome sequence data). The estimated single chromosome sizes for 1154–74 (O49) and 10432–62 (O27) are 3,889,393 bps and 3,801,481 bps and respectively, compared to the overall combined average of Chr I and Chr II of 4,077, 084 bps for all strains. The different enzyme maps for the two isolates are all consistent with one another and all maps indicate a single chromosome rather than the usual two chromosomes found in *V*. *cholerae*.

Recently, construction and characterization of genetically engineered *V*. *cholerae* strains with various chromosomal configurations including one with a single chromosome were reported [12]. Further, these authors have identified the genetic factors and topological requirements that are critical for stable replication and maintenance of single chromosome containing *V*. *cholerae* strains [12,26]. Naturally occurring single chromosome containing *V*. *cholerae* strains reported here present opportunities to address and validate these findings as well as address questions on how multiple origins present on a single chromosome are regulated to ensure faithful replication and partitioning.

### Verification of the presence of single chromosomes in *V*. *cholerae* strains 1154–74 (O49) and 10432–62 (O27) by PFGE

**Fig 4:**
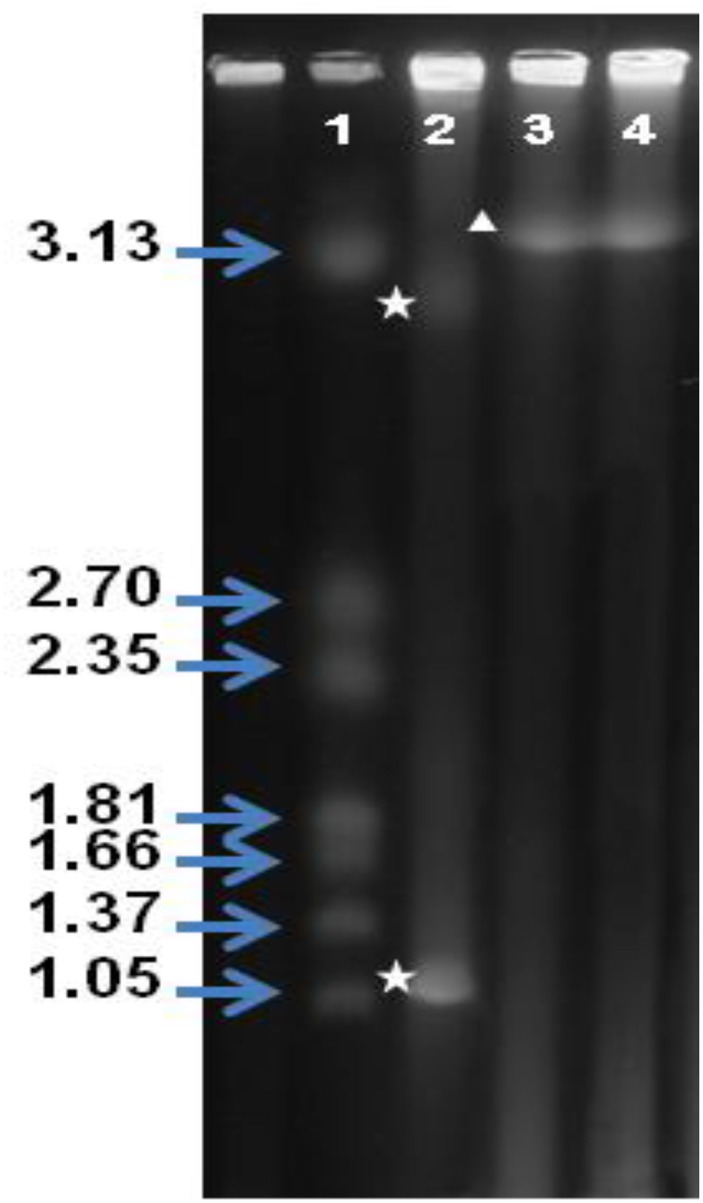
Pulse field gel electrophoresis of chromosomal DNAs of *V*. *cholerae* 1154–74 (O49) and 10432–62 (O27) strains. PFGE of intact *V*. *cholerae* DNA isolated from different *V*. *cholerae* strains. Lanes from left to right: 1) Molecular weight marker (Mbases) *H*. *wingeii* chromosomes, 2) *V*. *cholerae* O1 N16961 (the bands corresponding to Chr I and Chr II are marked by an asterisk), 3) *V*. *cholerae* 10432–62 (O27) and 4) *V*. *cholerae* 1154–74 (O49). In lanes 3 and 4, the band corresponding to the single chromosome is marked by a triangle.

In order to independently verify the presence of single chromosomes, whole genome analysis of intact chromosomes was done by PFGE (Fig. 4). In this analysis, *V*. *cholerae* O1 strain N16961, as expected showed two bands (~3Mb and ~1Mb) corresponding to Chr I and Chr II respectively [11], whereas 1154–74 (O49) and 10432–61 (O27) strains showed a single band >3 Mb in size (above the ~3.1 Mb marker). The resolution of PFGE in this size range was not very accurate under the electrophoresis conditions used and hence these are only estimated sizes of the chromosomes. Nonetheless, it corresponds well to the Whole Genome Mapping and whole genome sequencing (unpublished) based size estimates and provides further evidence for the observed single chromosome configuration in these strains.

### Comparative Whole Genome Map analysis and identification of genomic variations: identification of strains with putative tandem duplications

**Fig 5:**
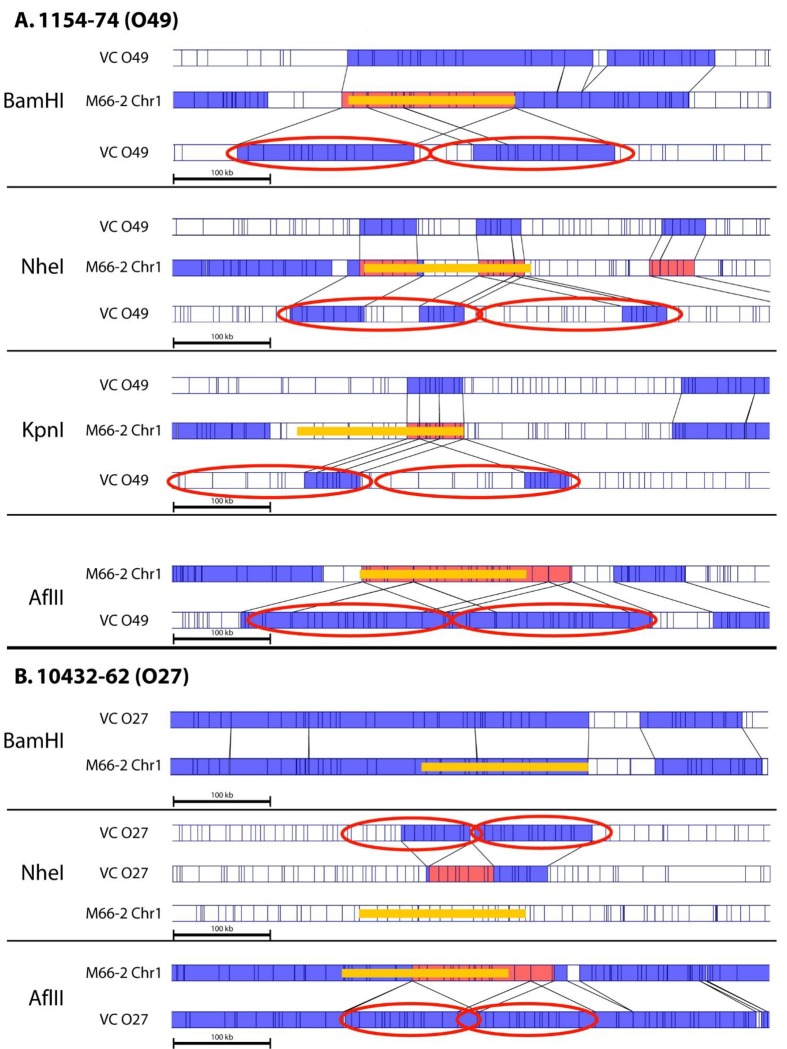
Whole Genome Maps using different restriction enzymes show putative tandem duplication of chromosomal regions. A: Top four panels of maps generated using *Bam*HI, *Nhe*I, *Kpn*I and *Afl*III respectively show the duplicated region in *V*. *cholerae* 1154–74 (O49). B: The bottom three panels of maps are that of 10432–62 (O27) using *Bam*HI, *Nhe*I and *Afl*III. In both cases, the location of duplication was found to be around 1240 kb to 1506 kb on reference M66–2 Chr I. The duplicated genome segments are indicated by the orange box in the *in silico* map of the reference strain. In some panels both assemblies with the single and two copies (resulting from re-analyses of the single molecule data from this region) are shown. The duplicated copies where they could be resolved are indicated by the red circles. The exact lengths of the duplications in the two strains cannot be unequivocally determined by this WGM data. It is also possible that the maps represent a mixed population of cells containing single and two copies of the duplication. (Scale bar 100 Kb).

In addition to the genome fusions, for both single chromosome isolates, 1154–74 (O49) and 10432–62 (O27), there is evidence of a putative tandem duplication event (~160 Kb) around the region spanning 1240 Kb and 1506 Kb of the reference genome (M66–2) sequence. From a comparison to the *in silico* map of M66–2, these suspected regions appear in the same location relative to M66–2 even though the two strains have very different overall chromosomal architecture. To aid visually, the orange box in the *in silico* maps (Fig. 5A) corresponds to the duplicated region (1.30 Mb to 1.47 Mb of the reference genome). In some panels the original single copy map with anomalous assemblies are shown. Upon reanalysis of the single molecule map data from this region, the two copies were resolved in strain 1154–74 (O49) and the resulting two copies are indicated by the red circles in the rearranged maps (Fig. 5A).

Whole Genome Maps of the same region in 10432–62 (O27) using various enzymes are shown in Fig. 5B. However, in this strain, the *Bam*HI Whole Genome Map did not resolve the two copies and hence shows only a single copy of the duplicated region whereas *Nhe*I and *Afl*II maps resolved the two copies of this region. Again, in this case, the orange box indicates the duplicated region around 1.30 Mb to 1.47 Mb of the reference genome and the red circles indicate the duplicated copies (Fig. 5B).

Examination of other *V*. *cholerae* maps revealed no evidence for this type of duplication in any other strain. There appears to be less clear evidence of the duplication in 10432–62 (O27) compared to 1154–74 (O49); nonetheless, we report this duplication based on WGMs generated using some enzymes. As this apparent duplication appears across multiple enzymes and in two strains at the same genomic location, it is highly unlikely that it is a mapping artifact. However, this observation does not preclude the possibility of a mixed population of two genetic variants; i.e., each bacterial cell has one or the other copy of the region, rather than having tandem duplication in a homogenous population.

### Comparative Whole Genome Map analysis and identification of genomic variations: identification of insertions, deletions and other large scale rearrangements

**Fig 6:**
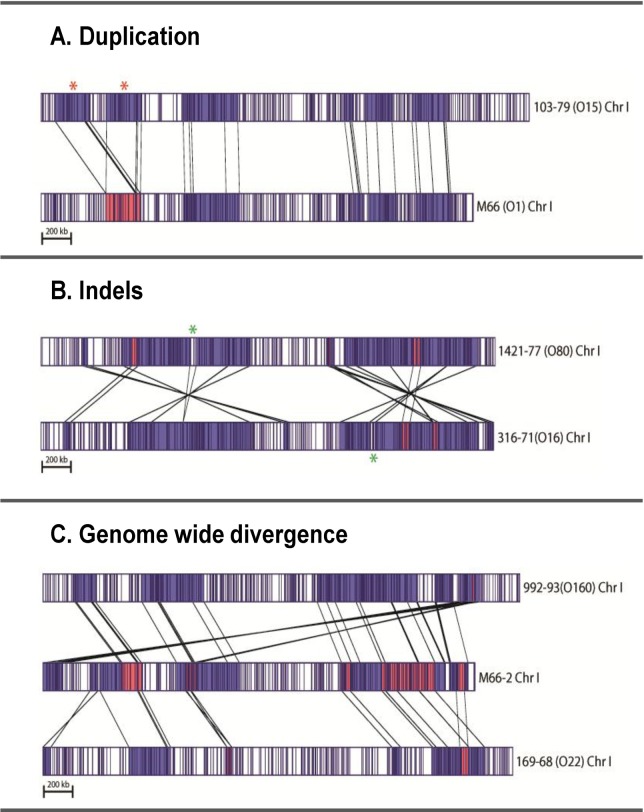
Whole Genome Mapping data using different restriction enzymes support various chromosomal rearrangements. Large scale chromosomal rearrangements deciphered by comparative analyses of Whole Genome Maps of Chr I in respective strains are indicated. Panel A: Whole Genome Maps of M66–2 compared to 103–79 (O15) indicating duplications of ~230 kb (red asterisks); Panel B: Whole Genome Maps of 1421–77 (O80) compared to 316–71 (O16) indicating indels of ~40 kb (green asterisks); Panel C: Whole Genome Maps of 992–92 (O160) and 169–68 (O22) compared to *in silico* map of M66–2 showing overall whole genome dissimilarity indicated by the white regions, interspersed with some segments of homology (dark blue color). The blue color indicates regions of single copy matches and the red color corresponds to matches with two copies. (Scale bar 200 Kb).

Compared to the sequence based size estimate of a reference genome such as M66–2, Chr I of 75 strains mapped in this study showed increase in size (putative insertions) ranging anywhere from 1,643 bps to 375,757 bps and 14 strains showed decrease in size (putative deletions) ranging from 4,839 bps to 233,146 bps (Table 1 and S1 Table). With respect to Chr II, 61 strains showed increase in size (putative insertions) ranging from 4,634 bps to 312,449 bps and 28 strains showed decrease in size (putative deletions) ranging from 255 bps to 87,752 bps (Table 1 and S1 Table). In order to assess whether these differences are significant, Whole Genome Map comparisons were performed, and some highlights are presented here. A comparison of the *in silico* map of Chr I of M66–2 to 103–79 (O15; estimated insertion of 375,757 bps) revealed duplicated regions (approximately 230 kb) in strain 103–79 (O15) as compared to M66–2 (Fig. 6A). Additionally, comparisons of more similar strains were performed (similarity based on Whole Genome Map clustering), to see if indels could be detected in these strains. For example, Whole Genome Maps of Chr I of strains 1421–77 (O80; estimated increase in size of 147,940 bps) and 316–71 (O16; estimated increase in size of 137,171 bps) were compared, and indels (approximately 40 kb) were found (Fig. 6B). Thus, despite limitations in precisely determining chromosome length by Whole Genome Mapping some of the large insertions, deletions and duplications were found to be significant and may account for the overall size differences observed. On the other hand, comparison of Chr I of M66–2 to that of 992–93 (O160) (increase in size of 300,293 bps) and 169–68 (O22) (increase in size of 253,155 bps) indicated that the size difference is not accounted for by simple insertions or deletions but overall genome wide divergence between the strains compared (Fig. 6C) and as expected these strains are far removed from each other in the dendrogram (Fig. 2).

## Conclusions

This study provides a snapshot of the genomic complexities that are prevalent in and population genetic diversity among non-O1/non-O139 *V*. *cholerae* strains and also reports on the discovery of novel, naturally occurring *V*. *cholerae* with single chromosomes. It is worthwhile to note that these rearrangements (insertions, deletions and duplications) probably occur frequently at high rates and can be isolated fortuitously. For example, Reams et al [27] reported that duplications arise in *Salmonella enterica* cultures at a rate of 10^–3^–10^–5^/cell/division and consequently the derivatives containing such aberrant chromosomal structures may not be representative of the original population from which they were derived. The single chromosome *V*. *cholerae* strains discovered in this study raise interesting biological questions on the mechanisms of chromosome replication, maintenance and partitioning in *V*. *cholerae* and other organisms that carry a single chromosome but more than one origin of replication. Given the recent revolution in next generation sequencing technologies, the Sakazaki strain collection would be an ideal collection on which to apply whole genome sequencing to understand the genetic potential and genomic diversity at the sequence level.

## Supporting Information

S1 Table Features of Sakazaki O-serogroup reference strains of *Vibrio cholerae*. (PDF)

S1 Dataset Chr_I_cluster_v6.xml: WGM data of chromosome I used to generate Fig. 2. (ZIP)

S2 Dataset Chr_II_cluster_v6.xml: WGM data of chromosome I used to generate Fig. 2. (ZIP)
