# Supplementary material for: Scanning the Landscape of Genome Architecture of Non-O1 and Non-O139 Vibrio cholerae by Whole Genome Mapping Reveals Extensive Population Genetic Diversity
Source: PLoS One. 2015 Mar 20;10(3):e0120311. doi: 10.1371/journal.pone.0120311 (PMC4368569; doi:10.1371/journal.pone.0120311)
Supplement: S1 Table — (PDF) [file pone.0120311.s001.pdf]

S1 Table: Features of Sakasaki O-serogroup reference strains of *Vibrio cholerae*						Estimated size (bp)					
O-serogroup	Reference Strain	Source	Country	Year	Optical Map	Chr I	M66-2	Insertion/Deletion	Chr II	M66-22	Insertion/Deletion2
Ol(Inaba)	NIH35A3*		Kasauli**	1941	Yes	3053161	2892523	-160638	1107232	1046382	-60850
O2	NCTC4711				Yes	2942741	2892523	-50218	1126558	1046382	-80176
O3	NCTC4715				Yes	3127779	2892523	-235256	1051016	1046382	-4634
O4	NCTC4716				Yes	2912565	2892523	-20042	1070689	1046382	-24307
O5	B4202-64	Diarrhea	Philippines	1964	Yes	2930489	2892523	-37966	1043229	1046382	3153
O6	7007-62	Diarrhea	India	1962	Yes	2885370	2892523	7153	993583	1046382	52799
O7	8394-62	Diarrhea	India	1962	Yes	2942236	2892523	-49713	1041478	1046382	4904
O8	10317-62	Diarrhea	Philippines	1962	Yes	3090590	2892523	-198067	1257292	1046382	-210910
O9	112-68	Diarrhea	Philippines	1968	Yes	2998320	2892523	-105797	1042940	1046382	3442
O10	218-68	Diarrhea	India	1968	Yes	3044120	2892523	-151597	1232769	1046382	-186387
O11	10843-62	Diarrhea	India	1962	Yes	2978153	2892523	-85630	1076529	1046382	-30147
O12	211-72	Diarrhea	India	1972	Yes	2950768	2892523	-58245	1183054	1046382	-136672
O13	11416-62	Diarrhea	Philippines	1962	Yes	3162859	2892523	-270336	1108268	1046382	-61886
O14	B8645-64	Diarrhea	India	1964	Yes	2928440	2892523	-35917	1086142	1046382	-39760
O15	103-79	Diarrhea	India	1979	Yes	3268280	2892523	-375757	1027005	1046382	19377
O16	316-71	Diarrhea	India	1971	Yes	3029694	2892523	-137171	1123322	1046382	-76940
O17	110-68	Diarrhea	India	1968	Yes	2934605	2892523	-42082	1071932	1046382	-25550
O18	B5257-64	Diarrhea	India	1964	Yes	2859044	2892523	33479	1077168	1046382	-30786
O19	139-68	Diarrhea	India	1968	Yes	2917912	2892523	-25389	1310878	1046382	-264496
O20	10332-62	Diarrhea	India	1962	Yes	3078965	2892523	-186442	1214057	1046382	-167675
O21	109-68	Diarrhea	India	1968	Yes	3036156	2892523	-143633	1174965	1046382	-128583
O22	169-68	Diarrhea	Philippines	1968	Yes	3145678	2892523	-253155	1243702	1046382	-197320
O23	317-71	Diarrhea	India	1971	Yes	2914308	2892523	-21785	1126089	1046382	-79707
O24	14438-62	Diarrhea	Philippines	1962	Yes	3039447	2892523	-146924	1060099	1046382	-13717
O25	14821-62	Diarrhea	India	1962	Yes	2825141	2892523	67382	1127927	1046382	-81545
O26	334-72	Diarrhea	Philippines	1972	Yes	2919692	2892523	-27169	998468	1046382	47914
O27	10432-62	Diarrhea	Philippines	1962	Yes	3801481	2892523	-908958	3801481	1046382	-2755099
O28	12530-62	Diarrhea	Philippines	1962	Yes	2831933	2892523	60590	1078513	1046382	-32131
O29	161-68	Diarrhea	India	1968	Yes	2858340	2892523	34183	1063299	1046382	-16917
O30	12795-62	Diarrhea	Philippines	1962	Yes	3064455	2892523	-171932	1311769	1046382	-265387
O31	5473-62	Diarrhea	Philippines	1962	Yes	2952235	2892523	-59712	1002570	1046382	43812
O32	171-68	Diarrhea	India	1968	Yes	2976682	2892523	-84159	1358831	1046382	-312449
O33	151-68	Diarrhea	India	1968	Yes	2964755	2892523	-72232	970752	1046382	75630
O35	1311-69	Diarrhea	India	1969	Yes	2903057	2892523	-10534	1045844	1046382	538
O36	1321-69	Diarrhea	Philippines	1969	Yes	2954572	2892523	-62049	994115	1046382	52267
O37	1322-69*	Diarrhea	India	1969	Yes	3005144	2892523	-112621	1069047	1046382	-22665
O38	215-68	Diarrhea	India	1968	Yes	2811409	2892523	81114	990630	1046382	55752
O41	284-73	Diarrhea	India	1973	Yes	2798891	2892523	93632	1225096	1046382	-178714
O42	284-73	Diarrhea	India	1973	Yes	2907583	2892523	-15060	1045417	1046382	965
O43	104-73	Diarrhea	India	1973	Yes	2910444	2892523	-17921	1098578	1046382	-52196
O44	112-73	Diarrhea	India	1973	Yes	2852268	2892523	40255	1120203	1046382	-73821
O45	122-73	Diarrhea	India	1973	Yes	3007986	2892523	-115463	1079608	1046382	-33226
O46	128-73	Diarrhea	India	1973	Yes	3022714	2892523	-130191	1074018	1046382	-27636
O47	131-73	Diarrhea	India	1973	Yes	2887684	2892523	4839	958630	1046382	87752
O48	133-73	Diarrhea	India	1973	Yes	2972906	2892523	-80383	1025202	1046382	21180
O49	1154-74	Diarrhea	India	1974	Yes	3889393	2892523	-996870	3889393	1046382	-2843011
O50	190-75	Diarrhea	India	1975	Yes	3059749	2892523	-167226	1068562	1046382	-22180
O51	198-73	Diarrhea	India	1973	Yes	3083782	2892523	-191259	1046127	1046382	255
O52	207-73	Diarrhea	India	1973	Yes	2862452	2892523	30071	1009802	1046382	36580
O53	1157-74	Diarrhea	India	1974	Yes	3056137	2892523	-163614	981854	1046382	64528
O54	1175-74	Diarrhea	India	1974	Yes	3087797	2892523	-195274	1103559	1046382	-57177
O58	1162-74	Diarrhea	India	1974	Yes	2913073	2892523	-20550	1092444	1046382	-46062
O67	121-79	Diarrhea	India	1979	Yes	2659377	2892523	233146	1018931	1046382	27451
O68	293-78	Sea Water	Japan	1978	Yes	2933764	2892523	-41241	1016196	1046382	30186
O71	162-78	Bird	Denmark	1978	Yes	2951774	2892523	-59251	1332780	1046382	-286398
O73	113-79	Seafish	Japan	1979	Yes	2884088	2892523	8435	1081651	1046382	-35269

O76	1158-76	Sea Water	Japan	1976	Yes	2917513	2892523	-24990	1114648	1046382	-68266
O77	8-76	Diarrhea	India	1976	Yes	3026334	2892523	-133811	1087333	1046382	-40951
O80	1421-77	Diarrhea	India	1977	Yes	3040463	2892523	-147940	1001818	1046382	44564
O81	318-78	Diarrhea	India	1978	Yes	2926155	2892523	-33632	1022728	1046382	23654
O83	1042-78	Diarrhea	USA	1978	Yes	3149673	2892523	-257150	1096222	1046382	-49840
O85	1903-83	Diarrhea	Jordan	1983	Yes	2835705	2892523	56818	1117555	1046382	-71173
O88	748-80	Diarrhea	USA	1980	Yes	2982964	2892523	-90441	1103109	1046382	-56727
O89	984-81	Diarrhea	India	1981	Yes	3035163	2892523	-142640	992020	1046382	54362
O90	1457-78	Diarrhea	Japan	1978	Yes	3001395	2892523	-108872	1109857	1046382	-63475
O92	NU193	Sewage	Japan	1987	Yes	2925328	2892523	-32805	972258	1046382	74124
O99	554-88	River Water	China	1988	Yes	2945592	2892523	-53069	1134240	1046382	-87858
O100	558-88	River Water	China	1988	Yes	2899224	2892523	-6701	1155610	1046382	-109228
O101	559-88	Diarrhea	China	1988	Yes	3073453	2892523	-180930	1222252	1046382	-175870
O102	563-88	Diarrhea	China	1988	Yes	2866245	2892523	26278	1123277	1046382	-76895
O103	567-88	Diarrhea	China	1988	Yes	3169405	2892523	-276882	1091345	1046382	-44963
O104	570-88	Diarrhea	China	1988	Yes	3045470	2892523	-152947	1059190	1046382	-12808
O105	571-88*	Diarrhea	China	1988	Yes	2984609	2892523	-92086	1086539	1046382	-40157
O106	572-88	Diarrhea	China	1988	Yes	2952927	2892523	-60404	1146764	1046382	-100382
O107	AU112	Rat	Japan	1989	Yes	3198909	2892523	-306386	1028450	1046382	17932
O108	AU124	Rat	Japan	1989	Yes	2984500	2892523	-91977	996995	1046382	49387
O109	AU165	Rat	Japan	1989	Yes	2937580	2892523	-45057	1111392	1046382	-65010
O110	AU256	Rat	Japan	1989	Yes	2964451	2892523	-71928	1178619	1046382	-132237
O111	AU291	Rat	Japan	1989	Yes	2939598	2892523	-47075	1114482	1046382	-68100
O112	AUR10	Rat	Japan	1989	Yes	2991003	2892523	-98480	1097408	1046382	-51026
O113	AU105	Rat	Japan	1989	Yes	2904356	2892523	-11833	1073274	1046382	-26892
O115	523-80	Diarrhea	USA	1980	Yes	2934259	2892523	-41736	1296434	1046382	-250052
O120	686-91	Diarrhea	Japan	1991	Yes	3204231	2892523	-311708	1070842	1046382	-24460
O121	555-80	Diarrhea	USA	1980	Yes	2949530	2892523	-57007	1069567	1046382	-23185
O122	1133-80	Diarrhea	Rumania	1980	Yes	3051106	2892523	-158583	1118918	1046382	-72536
O127	501-90	River Water	Japan	1990	Yes	3091133	2892523	-198610	1080791	1046382	-34409
O139 (Bengal)	63-93 (MO45)*	Diarrhea	India	1992	Yes	3077167	2892523	-184644	1034255	1046382	12127
O141	234-93*	Diarrhea	India	1993	Yes	3098243	2892523	-205720	1044344	1046382	2038
O144	254-93	Diarrhea	India	1993	Yes	3046849	2892523	-154326	1034639	1046382	11743
O154	968-93	Diarrhea	Indonesia	1993	Yes	2894166	2892523	-1643	1183994	1046382	-137612
O160	992-93	Diarrhea	Indonesia	1993	Yes	3192816	2892523	-300293	1119766	1046382	-73384
O34	152-68	Diarrhea	India	1968				0			0
O39	225-68	Diarrhea	India	1968				0			0
O60	195-75	Diarrhea	India	1975				0			0
O61	12-74	Diarrhea	India	1974				0			0
O69	1861-79	Diarrhea	India	1979				0			0
Ol(Ogawa)	NIH41*		India	1941				0			0
O40	212-72	Diarrhea	Japan	1972				0			0
O55	197-75	Diarrhea	India	1975				0			0
O56	475-75	Diarrhea	India	1975				0			0
O57	1463-76	Bird	Denmark	1976				0			0
O59	1333-74	Diarrhea	India	1974				0			0
O62	1-76	Diarrhea	India	1976				0			0
O63	19-76	Diarrhea	India	1976				0			0
O64	1280-75	Diarrhea	India	1975				0			0
O65	981-75	Diarrhea	India	1975				0			0
O66	993-75	Diarrhea	India	1975				0			0
O70	1111-77	Diarrhea	India	1977				0			0
O72	431-79	Diarrhea	India	1979				0			0
O74	428-79	Diarrhea	India	1979				0			0
O75	429-79	Diarrhea	India	1979				0			0
O78	27-76	Diarrhea	India	1976				0			0
O79	1103-76	Diarrhea	India	1976				0			0
O82	355-80	Diarrhea	India	1980				0			0

O84	840-83	Diarrhea	India	1983				0			0
O86	571-81	Diarrhea	Philippines	1981				0			0
O87	973-81	Diarrhea	India	1981				0			0
O91	796-80	Diarrhea	India	1980				0			0
O93	S5046	Diarrhea	India	1976				0			0
O94	S5o69	Diarrhea	India	1976				0			0
O95	S5697	Diarrhea	India	1976				0			0
O96	S6535	Diarrhea	India	1976				0			0
O97	S7443	Diarrhea	India	1976				0			0
O98	S6541	Diarrhea	India	1976				0			0
O114	246-79	River Water	USA	1979				0			0
O116	980-78	Sea Water	USA	1978				0			0
O117	381-82	River Water	Japan	1982				0			0
O118	58-91	Diarrhea	Israel	1991				0			0
O119	353-81	Diarrhea	India	1981				0			0
O123	345-81	Diarrhea	India	1981				0			0
O124	355-81	Diarrhea	India	1981				0			0
O125	436-81	Diarrhea	India	1981				0			0
O126	472-81	Diarrhea	India	1981				0			0
O128	819-87	Diarrhea	Japan	1987				0			0
O129	420-81	Diarrhea	India	1981				0			0
O130	447-81	Diarrhea	India	1981				0			0
O131	813-91	Diarrhea	India	1991				0			0
O132	767-81	Diarrhea	Thailand	1981				0			0
O133	816-91	Diarrhea	India	1991				0			0
O134	821-91	Diarrhea	India	1991				0			0
O135	29-92	Diarrhea	India	1992				0			0
O136	YCH11	Shellfish	Japan	1992				0			0
O137	448-92	Blood	Japan	1992				0			0
O138	455-92	Crab	Japan	1992				0			0
O140 (Hakata)	487-85	Sea Water	Japan	1985				0			0
O142	411-93	Diarrhea	Argentina	1993				0			0
O143	242-93	Diarrhea	India	1993				0			0
O145	255-93	Diarrhea	India	1993				0			0
O146	258-93	Diarrhea	India	1993				0			0
O147	259-93	Diarrhea	India	1993				0			0
O148	322-93	Diarrhea	Indonesia	1993				0			0
O149	327-93	Diarrhea	Indonesia	1993				0			0
O150	504-93	Diarrhea	Indonesia	1993				0			0
O151	511-93	Diarrhea	Indonesia	1993				0			0
O152	957-93	Diarrhea	Indonesia	1993				0			0
O153	966-93	Diarrhea	Indonesia	1993				0			0
O155	490-93	Diarrhea	Thailand	1993				0			0
O156	975-93	Diarrhea	Indonesia	1993				0			0
O157	977-93	Diarrhea	Indonesia	1993				0			0
O158	985-93	Diarrhea	Indonesia	1993				0			0
O159	989-93	Diarrhea	Indonesia	1993				0			0
O161	1003-93	Diarrhea	Indonesia	1993				0			0
O162	936-93	Diarrhea	Argentina	1993				0			0
O163	1093-93	Diarrhea	Germany	1993				0			0
O164	1182-93	Diarrhea	Indonesia	1993				0			0
O165	1187-93	Diarrhea	Indonesia	1993				0			0
O166	1215-93	Diarrhea	Indonesia	1993				0			0
O167	356-93	Diarrhea	India	1993				0			0
O168	NQ-44	Diarrhea	Japan	1991				0			0
O169	NQ-49	Diarrhea	Japan	1991				0			0
O170	NQ-78	Diarrhea	Japan	1991				0			0
O171	NQ-158	Diarrhea	Japan	1992				0			0

O172	NQ-163	Diarrhea	Japan	1992				0			0
O173	NQ-191	Diarrhea	Japan	1992				0			0
O174	3006-94	Diarrhea	Bangladesh	1994				0			0
O175	287-94	Diarrhea	Brazil	1994				0			0
O176	337-94	Diarrhea	India	1994				0			0
O177	658-94	Environmen	Korea	1994				0			0
O178	773-94	Diarrhea	India	1994				0			0
O179	863-94	Frozen Praw	Denmark	1994				0			0
O180	1223-93	Diarrhea	Indonesia	1993				0			0
O181	772-94	Diarrhea	India	1994				0			0
O182	263-95	Diarrhea	Indonesia	1995				0			0
O183	297-95	Diarrhea	Thailand	1995				0			0
O184	352-95	Diarrhea	Brazil	1995				0			0
O185	366-95	Diarrhea	Brazil	1995				0			0
O186	372-95	Frozen Praw	Denmark	1995				0			0
O187	626-95	Frozen Praw	Germany	1995				0			0
O188	1311-95	River Water	Korea	1995				0			0
O189	326-96	Diarrhea	Brazil	1996				0			0
O190	363-96	Diarrhea	India	1996				0			0
O191	366-96*	rozen Seafis	Japan	1996				0			0
O192	1092-96	Diarrhea	India	1996				0			0
O193	1234-96	Diarrhea	Thailand	1996				0			0
O194***	621-98 (N-57)	Pond Water	Bangladesh	1998				0			0
O195	645-97	Water Fish	Germany	1997				0			0
O196	361-98	Diarrhea	India	1998				0			0
O197	376-98	Diarrhea	India	1998				0			0
O198	463-98	Ram	Netherlands	1998				0			0
O199	756-97	Environmen	Peru	1997				0			0
O200	815-98	Diarrhea	India	1998				0			0
O201	667-96	Environmen	Japan	1996				0			0
O202	738-96	Diarrhea	Japan	1996				0			0
O203	4022-98	Diarrhea	India	1998				0			0
O204	1188-98	Shrimp	Germany	1998				0			0
O205	501-99	Diarrhea	India	1999				0			0
O206	118-2000	Diarrhea	India	2000				0			0
*CT- Positive Strain											
**From Central Research Institute, Kasauli											
*** Ansaruzzaman M, Shimada T, Bhuiyan NA, Nahar S, Alam K, Islam MS, Albert MJ. 1999. Cross-reaction between a strain of Vibrio mimicus											
and V. cholerae O139 Bengal. J Med Microbiol. Sep;48(9):873-7.											
	Vibrio mimicus										
	Single chromosome *V. cholerae*										
-:inserion; + deletion compared to the reference genome											
